# Targeting Obesity-Associated Breast Cancer: Curcumin Counteracts Leptin-Induced Tumorigenic and Metastatic Traits in MCF-7 and MDA-MB-231 Breast Cancer Cells

**DOI:** 10.3390/ijms27198572

**Published:** 2026-09-25

**Authors:** Hanène Attia, Orace Mathieu Kenou, Khouloud Ayed, Stanley Arinze Abonyi, Rym Akrout, Rahma Boughriba, Ali Ben Cheikh, Sylvère Fidèle Fassinou, Tomabu Adjobimey, Wassim Y. Almawi, Asma Gati

**Affiliations:** 1 Laboratory of Genetics, Immunology, and Human Pathologies, Faculty of Sciences of Tunis, University Tunis El Manar, Tunis 1068, Tunisia; 2 Laboratoire de Biologie Intégrative pour l’Innovation Thérapeutique (BioInov), Faculté des Sciences et Techniques (FAST), Université d’Abomey-Calavi, Abomey-Calavi BP 526, Benin; 3 Faculty of Pharmaceutical Sciences, University of Nigeria, Nsukka 410105, Nigeria

**Keywords:** obesity, breast cancer, hyperleptinemia, curcumin, nutraceutical, tumorigenic traits, leptin signaling

## Abstract

Obesity-driven hyperleptinemia is a key driver of breast cancer (BC) aggressiveness; nevertheless, strategies to counteract leptin-mediated tumor progression remain limited. Curcumin, a natural polyphenol with established anti-obesity, anti-inflammatory, and anticancer properties, has emerged as a promising metabolic and tumor-modulating biomolecule, although its effects on leptin-induced oncogenic signaling remain unclear. This study evaluated Curcumin as an adjuvant nutraceutical against leptin-induced oncogenesis in MCF-7 and MDA-MB-231 cells. Cells were exposed to obesity-mimicking leptin levels alone or with non-toxic Curcumin doses. MTT, clonogenic, wound-healing, and 3D spheroid dissemination assays were used to assess phenotypic changes, while network pharmacology and molecular docking explored underlying mechanisms. Curcumin significantly inhibited leptin-induced proliferation (8–17%), colony formation (60–90%), migration (20–45%), and 3D spheroid dissemination (20%) in both BC cell lines. Network pharmacology identified shared hub proteins, including AKT1, RHOA, MAP2K1, and MAPK14, linking Curcumin targets with leptin-driven BC signatures, and molecular docking supported direct interactions of Curcumin with these proteins. These findings suggest that Curcumin counteracts hyperleptinemia-induced tumor progression by targeting the PI3K/AKT, MAPK/ERK, and RHOA/ROCK pathways. This integrated in vitro and in silico study supports Curcumin as a promising adjuvant nutraceutical for obesity-associated BC, although experimental pathway validation is still required.

## 1. Introduction

Obesity has evolved from a metabolic comorbidity into a critical driver of the global BC epidemic. With about 2.3 million new cases in 2020 (nearly one in four cancers), BC is the most diagnosed cancer in women, surpassing lung cancer in incidence and causing approximately 685.000 deaths (one in six cancer-related deaths globally). While screening and targeted therapies have reduced overall mortality, obesity-associated BC presents a distinct clinical challenge, characterized by increased incidence in postmenopausal women and a significantly higher risk of metastasis and chemoresistance. Growing evidence suggests that obesity promotes tumor initiation and progression through interconnected processes such as chronic low-grade inflammation, insulin resistance, altered estrogen metabolism, and dysregulated adipokine signaling [1,2,3,4,5,6].

Central to this obesity–cancer axis is the adipokine leptin, a 16 kDa peptide hormone encoded by the LEP gene and primarily secreted by adipose tissue. While its classical role is to regulate the energy balance via the hypothalamus, it also acts as a pleiotropic signaling molecule that modulates inflammation, immunity, and cellular growth, particularly in mammary epithelial cells. Circulating leptin levels increase proportionally with adipose tissue mass, leading to hyperleptinemia in obese individuals [1,3,4,5,7,8]. Chronic hyperleptinemia in obese individuals facilitates oncogenesis through the overactivation of the leptin receptor (LepRb) and the activation of JAK2/STAT3, PI3K/AKT, and MAPK/ERK pathways, promoting tumor cell proliferation, metastasis, and chemoresistance, while also supporting mammary stem cell survival and contributing to vascular permeability and tumor angiogenesis. Furthermore, leptin modulates the tumor microenvironment, influencing the anti-tumor immune response and enhancing malignant cell growth [9,10,11,12]. Significant efforts have been directed toward developing therapeutic strategies interfering with leptin signaling and its downstream oncogenic effects, including the use of peptide antagonists, monoclonal antibodies, and leptin-neutralizing agents [5,7,12,13,14,15,16,17,18,19,20,21,22,23]. However, these direct approaches face significant limitations, as systemic inhibition of leptin may induce adverse metabolic effects such as hyperphagia, weight gain, and insulin dysregulation [24,25].

The clinical imperative to neutralize leptin-driven oncogenesis without compromising systemic energy balance has shifted focus toward adjuvant nutraceuticals, compounds capable of uncoupling metabolic dysfunction from tumor progression. Among these, Curcumin (1,7-bis(4-hydroxy-3-methoxyphenyl)-1,6-heptadiene-3,5-dione), the primary bioactive polyphenol derived from Curcuma longa, has emerged as a frontline candidate due to its dual-regulatory capacity. Curcumin’s relevance to obesity-associated BC stems from its metabolic-oncogenic intersectionality. Besides its antioxidant, anti-inflammatory, and antimicrobial effects, Curcumin plays a key role in metabolic regulation, improving glucose and lipid metabolism, enhancing insulin sensitivity, and contributing to overall energy homeostasis, while reducing obesity-associated inflammation and dyslipidemia [26,27,28]. This therapeutic potential of Curcumin extends beyond simple metabolic correction, as it functions as a potent anticancer agent by directly intercepting the intracellular signaling cascades that leptin targets. Specifically, Curcumin exerts pleiotropic inhibitory effects on NF-κB, STAT3, and PI3K/AKT/mTOR signaling nodes that are chronically overactivated in the hyperleptinemic state [29,30,31,32]. This dual action simultaneously addresses the metabolic dysregulation (the source of leptin) and the tumor’s proliferative response to that leptin (the downstream effect), leading to inhibition of cell proliferation, induction of apoptosis, and suppression of tumor growth and metastasis [33,34].

Despite its clear dual action, the specific efficacy of Curcumin in disrupting the leptin–LepRb axis remains under-characterized. It was largely restricted to hormone receptor-positive models, where Curcumin was found to attenuate leptin-induced growth in MCF-7 and T47D cells [35,36]. In particular, the impact of Curcumin on triple-negative BC (TNBC), the most aggressive BC subtype characterized by high basal inflammatory signaling and poor prognosis, remains virtually unexplored in the context of hyperleptinemia. Given that TNBC cells often exhibit a heightened sensitivity to adipokine-driven EMT, determining whether Curcumin can suppress leptin-mediated progression in this subtype is of urgent clinical interest.

Given this gap in the metabolic-oncologic framework, this study evaluated whether Curcumin attenuates leptin-induced proliferative and migratory oncogenic activity in MCF-7 (Luminal A) and MDA-MB-231 (TNBC) BC cell lines. To address the inherent complexity of Curcumin’s multi-target profile, we integrated a network pharmacology analysis. This in silico approach served as a predictive framework to orient the research, identifying putative hub genes that govern the Curcumin–leptin–BC axis, rather than providing definitive evidence of physical interaction. By leveraging these computational insights to guide and interpret the experimental findings, this study aims to refine our understanding of metabolic–oncogenic crosstalk and to evaluate Curcumin’s potential as a candidate for disrupting the signaling nexus in obesity-associated BC.

## 2. Results

### 2.1. Curcumin Impairs Hyperleptinemia-Driven Tumorigenic Phenotype of MCF-7 and MDA-MB-231 BC Cell Lines

#### 2.1.1. Curcumin Attenuates Leptin-Induced BC Cell Viability

To assess whether Curcumin could counteract leptin-driven viability, MCF-7 and MDA-MB-231 cells were treated with Curcumin (5, 10, 20, and 30 µM), leptin (100 ng/mL) alone or in combination with non-toxic doses of Curcumin (5 or 10 µM), and cell viability was evaluated using the MTT assay. As shown in Figure 1A, Curcumin induced a dose-dependent reduction in cell viability in both MCF-7 and MDA-MB-231 cells. However, treatment with 5 and 10 µM of Curcumin did not significantly affect cell viability, indicating that these concentrations did not exert substantial cytotoxic effects under the experimental conditions. Therefore, these two concentrations were selected for subsequent experiments investigating the effects of Curcumin on leptin-mediated responses. Leptin treatment alone (Figure 1B) increased the cell number in both MCF-7 and MDA-MB-231 cell lines by 20% (*p* < 0.0001) and (*p* < 0.01), respectively, relative to untreated cells. However, curcumin at 5 and 10 µM decreased leptin-induced cell proliferation by 8% and 16% (*p* < 0.001) in MCF-7 cells and by 12% (*p* < 0.05) and 17% (*p* < 0.01) in MDA-MB-231 cells.

#### 2.1.2. Curcumin Inhibits Leptin-Induced BC Cell Clonogenicity

The effect of Curcumin on leptin-induced clonogenic potential in MCF-7 and MDA-MB-231 cells was evaluated using a colony-forming assay. Cells were treated with medium, Curcumin alone (5 and 10 µM), leptin alone (100 ng/mL), or the combination of leptin and Curcumin for 48 h, followed by a 10-day incubation period.

As shown in Figure 2A, curcumin alone inhibited colony formation in both cell lines compared to the untreated control, reducing colony formation to 25.12% and 34.55% in MCF-7 cells, and to 77.88% and 18.84% in MDA-MB-231 cells, at 5 and 10 µM, respectively.

Leptin treatment (100 ng/mL) increased the percentage of colony formation relative to untreated controls by 20% (*p* < 0.05) in MCF-7 cells and by 22% (*p* < 0.05) in MDA-MB-231 cells (Figure 2B).

Co-treatment of MCF-7 cells with leptin and Curcumin significantly reduced colony formation compared with leptin alone, decreasing colony numbers to 19.4% and 28.27% of control at 5 and 10 µM, respectively (both at *p* < 0.0001). Similarly, in MDA-MB-231 cells, curcumin significantly attenuated leptin-induced colony formation in a dose-dependent manner. Treatment with 5 µM and 10 µM of Curcumin reduced colony formation to 39.89% and 9.62% (both at *p* < 0.0001), relative to leptin-treated cells.

#### 2.1.3. Curcumin Inhibits Leptin-Induced BC Cell Migration

The effect of Curcumin on leptin-induced migration was evaluated using a wound-healing assay.

As shown in Figure 3A, Curcumin significantly reduced migratory capacity in MCF-7 cells, yielding wound-closure percentages of 82.04% (*p* < 0.05) and 81.64% (*p* < 0.05) at 5 µM and 10 µM, respectively. In MDA-MB-231 cells, Curcumin at 5 µM reduced wound closure to 87.70%, while a more pronounced inhibition was observed at 10 µM, with migration decreasing to 55.65% (*p* < 0.001).

Representative images captured at 0 and 48 h (Figure 3B) illustrate the effects of different treatment conditions, namely untreated (control), leptin (100 ng/mL), leptin (100 ng/mL) + Curcumin (5 µM), and leptin (100 ng/mL) + Curcumin (10 µM) on gap closure in both cell lines. Quantitative analysis showed that leptin increased migration by 33% (*p* < 0.05) in MCF-7 cells and by 19% (*p* < 0.05) in MDA-MB-231 cells, when compared with untreated controls. However, the co-treatment of MCF-7 cells with leptin and Curcumin at 5 µM or 10 µM significantly reduced their migratory capacity compared to leptin-treated cells, yielding wound-healing percentages of 80.78% (*p* < 0.05) and 65.66% (*p* < 0.01), respectively. Similarly, in MDA-MB-231 cells, leptin-induced migration was strongly diminished when combined with Curcumin; co-treatment with 5 µM resulted in a wound-closure percentage of 68.31% (*p* < 0.05), whereas the combination with 10 µM produced a more significant decrease to 54.32% (*p* < 0.01), indicating robust inhibition of gap closure.

#### 2.1.4. Curcumin Decreases Leptin-Induced 3D Dissemination Potential of BC Spheroids

The invasive potential of MCF-7 and MDA-MB-231 cells treated with Curcumin (10 µM), Leptin (100 ng/mL), or their combination was evaluated using a spheroid dissemination assay and quantified by measuring the increase in spheroid area from baseline (T0 h) to 48 h post-stimulation (T48 h).

As presented in Figure 4A, treatment of MCF-7 and MDA-MB-231 cells with Curcumin alone at 10 µM significantly decreased dissemination to 88.22% (*p* < 0.05) and 70.03% (*p* < 0.05) of control levels, respectively.

Representative images (Figure 4B) captured at T0 and T48 h show that treatment with the non-toxic dose of Curcumin attenuated leptin-induced spheroid dissemination in both cell lines compared with that in the leptin-treated control group. When used alone, leptin increased spheroid dissemination by 21.5% (*p* < 0.05) in MCF-7 cells and 23% (*p* < 0.05) in MDA-MB-231 cells. As shown in Figure 4B, 10 µM of Curcumin significantly reduced the dissemination of MCF-7 and MDA-MB-231 spheroids to 75.44% (*p* < 0.05) and 54.27% (*p* < 0.01) of leptin-stimulated controls, respectively.

### 2.2. Network Pharmacology Workflow for Curcumin–Leptin–BC Cell Lines

#### 2.2.1. Identification of Common Targets Between Curcumin, Leptin, and BC Models; Protein–Protein Interaction (PPI) Network Construction and Hub Gene Identification

Putative molecular targets associated with Curcumin, leptin, and MCF-7 and MDA-MB-231 cells were retrieved according to the established relevance thresholds and were filtered to include only protein-coding genes. A total of 301 Curcumin-related targets were retrieved from PharmMapper in both cell line models, while 1466 leptin-related targets were retrieved overall. For the MCF-7 and MDA-MB-231 datasets, 2000 cell line-associated targets were obtained. The intersection analysis revealed 16 shared targets for MCF-7 (Figure 5A) and 19 shared targets for MDA-MB-231 (Figure 5B), indicating overlapping molecular components potentially affected by Curcumin and leptin. All common targets for each cell line were submitted to STRING using a minimum interaction confidence score of 0.4. For the MDA-MB-231 cell model, intersecting genes formed a fully connected PPI network consisting of 19 nodes and 68 edges, whereas the MCF-7 network consisted of 16 nodes and 37 edges. Topological analysis, performed in Cytoscape v3.10.4 prior to visualization, identified the most connected nodes as hub genes for each cell line. In the final PPI network visualization (Figure 5C,D), hub genes were displayed using ellipse-shaped nodes, whereas all other targets appeared as rectangles. For MCF-7, the identified hub genes were *AKT1*, *HSP90AA1*, *RHOA*, *CDC42*, *RAC1*, *B2M*, *PTPN1*, *STAT1*, *HPRT1*, *RHEB*, and *CTSB*. For MDA-MB-231, the hub genes were *AKT1*, *HSP90AA1*, *RHOA*, *MAP2K1*, *BCL2L1*, *CDC42*, *RAC1*, *MAPK14*, *PLAU*, *RHEB*, and *PTPN1*.

#### 2.2.2. Functional Enrichment

Biological processes for both cell lines are summarized in Figure 6. Functional enrichment analysis of MCF-7 hub genes showed that the most significantly enriched biological processes were the regulation of plasma membrane–bound cell projection organization and regulation of cell projection organization. These were driven by genes such as *CDC42*, *RAC1*, *AKT1*, *RHOA*, *HSP90AA1*, *B2M*, and *PTPN1,* with a –log FDR of 4.8. For the MDA-MB-231 dataset, the most enriched biological process was response to external stimulus, associated with *PLAU*, *RNASE1*, *RAC1*, *RHOA*, *HSP90AA1*, *MAPK14*, *AKT1*, *BCL2L1*, *CDC42*, *MAP2K1*, and *PTPN1,* yielding a −log FDR of 5.5, while additional processes such as regulation of plasma membrane–bound cell projection organization and regulation of cell projection organization were also observed with a −log FDR of 5.

#### 2.2.3. Molecular Docking, Validation and MM-GBSA Binding Energy Analysis

Molecular docking simulations were conducted to assess the interaction profile of Curcumin with the druggable protein targets encoded by the hub genes. All protein structures used met the resolution threshold of <2.5 Å. The docking results, including Glide docking scores and MM-GBSA binding free energies for each protein–ligand complex, are summarized in Table 1. Among the evaluated targets, MAPK14 (PDB: 6SFO) showed the most favorable predicted docking score (−10.800 kcal/mol), with an estimated MM-GBSA binding free energy of −63.10 kcal/mol. AKT1 (8UW7) and RHOA (1FTN) also showed favorable predicted docking scores of −8.285 and −7.107 kcal/mol, respectively, with corresponding estimated MM-GBSA binding free energies of −66.95 and −55.41 kcal/mol. Additional favorable predicted interactions were observed for MAP2K1 (8YP4), PTPN1 (1PTY), and RHEB (1XTQ), with docking scores of −6.445, −4.593, and −4.475 kcal/mol, respectively.

To validate the docking protocol, the cognate ligand of *MAPK14* (PDB: 6SFO), which has the most favorable docking score, was redocked into its binding pocket. The resulting superimposition yielded an RMSD of 0.5304 Å (Figure 7), indicating good reproduction of the experimentally observed ligand binding pose and supporting the reliability of the docking protocol. The ligand–protein complexes were visualized using Discovery Studio 2025 Client. The structural overlay of all the complexes is presented in Figure 8, with their 3D and 2D interactions.

## 3. Discussion

Adipokine-mediated signaling is increasingly recognized as a major determinant of BC progression within the tumor microenvironment, particularly in the context of obesity, where leptin acts as a key driver of tumor growth, survival, and aggressiveness. Curcumin, a polyphenolic compound with well-documented anti-obesity and antitumorigenic properties [37], has emerged as a promising candidate for targeting obesity-associated oncogenic pathways. However, the molecular interplay between leptin and Curcumin in BC remains largely unexplored. To address this gap, we evaluated Curcumin’s capacity to counteract leptin-induced tumorigenic traits in MCF-7 and MDA-MB-231 BC cell lines and applied an integrative network pharmacology approach to identify candidate mediators of their interaction. The mechanistic framework derived from these computational analyses is discussed as a hypothesis-generating construct, and all predicted interactions require experimental validation. Our findings demonstrate that exposure to a high leptin concentration (100 ng/mL), reflecting obesity-associated hyperleptinemia, significantly enhanced proliferation, clonogenicity, and migration in both cell lines, consistent with the established role of leptin in promoting aggressive tumor behavior [9,35,38,39].

In line with previously reported work, Curcumin alone exerted inhibitory effects across the evaluated phenotypic tumorigenic traits [33,35,40,41,42,43,44]; however, its suppressive effect was generally more pronounced when cells were co-treated with leptin. In particular, Curcumin markedly attenuated the leptin-induced increases in proliferation, clonogenicity, migration, and 3D spheroid dissemination, with the strongest effects observed at 10 µM. For instance, 10 µM Curcumin reduced colony formation to 28.27% and 9.62% in leptin-stimulated MCF-7 and MDA-MB-231 cells, respectively, compared with 34.55% and 18.84% following Curcumin alone. Similarly, spheroid dissemination decreased to 75.44% and 54.27% of the leptin-treated controls upon co-treatment, compared with 88.22% and 70.03% of untreated controls with Curcumin alone. Taken together, these findings indicate that Curcumin possesses intrinsic anti-tumor activity while displaying a particularly pronounced capacity to counteract the aggressive phenotype induced by leptin.

Inhibition of leptin-stimulated proliferation by Curcumin has previously been reported in MCF-7 [35] and T47D cells [36]. Our findings extend this effect to MDA-MB-231, a triple-negative, mesenchymal-like model. Furthermore, we report, to the best of our knowledge, for the first time, that Curcumin markedly suppresses leptin-induced clonogenic capacity in both cell lines. Mechanistically, while leptin promotes cell cycle progression by increasing the proportion of cells in S and G2/M phases [7], Curcumin induces cell cycle arrest at late S and G2/M phases through modulation of key cell cycle regulators, including CDC25 and CDC2 and p21 [32,45,46].

Beyond proliferation, Curcumin at non-cytotoxic concentrations significantly attenuated leptin-induced migration in both 2D wound-healing and 3D spheroid dissemination assays. At the mechanistic level, leptin-driven migration is substantially mediated by the induction of epithelial–mesenchymal transition (EMT) and the upregulation of matrix metalloproteinases (MMPs) [7,39]. However, curcumin is known to oppose migratory programs by restoring epithelial markers, downregulating mesenchymal markers and EMT-related transcription factors, and reducing MMP expression [32,46]. Although not assessed in the present study, these converging mechanisms are consistent with the migratory phenotypes we report and provide a framework for targeted mechanistic validation in future work.

To explore the molecular underpinnings of the observed phenotypic effects and to identify candidate mediators of the leptin-Curcumin crosstalk, we applied an integrative network pharmacology workflow. Six hub proteins, RHOA, AKT1, RHEB, MAP2K1, MAPK14, and PTPN1, were shared among Curcumin targets, leptin targets, and the molecular signatures of MCF-7 and MDA-MB-231 cells, with 16 and 19 overlapping targets identified per cell line, respectively. Molecular docking predicted putative interactions between Curcumin and each hub protein. While these in silico results require experimental validation, their convergence with published biochemical evidence provides a mechanistically coherent framework for interpreting our experimental observations. By synthesizing our in silico findings with established literature, we propose a mechanistic framework that elucidates leptin-Curcumin signaling crosstalk across the two BC cell lines (Figure 9), thereby providing a molecular basis for the phenotypic traits observed in our experimental data.

Leptin signaling is initiated through its binding to the leptin receptor (LepRb). Notably, Curcumin has been shown to downregulate leptin and leptin receptor gene expression, as well as leptin secretion, thereby directly dampening the leptin signaling loop [36]. Our group reported that LepRb is expressed on both MCF-7 and MDA-MB-231 BC cell surfaces, and leptin treatment induces its overexpression in MDA-MB-231 cells but not in MCF-7 cells [9]. A RHOA/ROCK/JAK2 signaling axis has been proposed downstream of LepRb, whereby leptin-induced activation of RHOA promotes ROCK recruitment and subsequent JAK2 activation [47,48,49].

RHOA emerged as a shared hub across both cell lines, with a favorable predicted docking score (−7.107 kcal/mol; ΔGbind: −55.41 kcal/mol). Together, these data support the hypothesis that Curcumin may disrupt leptin-driven oncogenic signaling partly through interference with RHOA-dependent pathways; direct experimental validation of this node is warranted.

JAK2 activation is known to orchestrate three major downstream pathways prominently enriched in our pharmacological network: PI3K/AKT/mTOR, MAPK/ERK, and JAK2/STAT3–STAT5 [2,7,48]. Within the PI3K/AKT axis, AKT1 emerged as the strongest computationally supported hub (docking score: −8.285 kcal/mol; ΔGbind of −66.95 kcal/mol). Dysregulation of the AKT/mTORC1/S6K cascade is a hallmark of BC and is associated with aggressive disease and poor prognosis [3,48,50,51]. Published evidence confirms that Curcumin inhibits AKT expression and phosphorylation in BC cells [35,51]. RHEB, a small GTPase that acts downstream of AKT as a direct activator of mTOR [51], was also identified as a shared hub protein in both cell lines. However, it exhibited a weaker predicted docking affinity (docking score: −4.475 kcal/mol; ΔGbind: −48.43 kcal/mol), and no published study has directly examined Curcumin’s effect on RHEB activity to date. Its identification as a shared hub protein nevertheless reinforces the centrality of the PI3K/AKT/mTOR network in mediating the leptin–Curcumin interplay and designates RHEB as a candidate node for future investigation.

Within the MAPK/ERK cascade, MAPK14 (p38 MAPK) and MAP2K1 (MEK1) were identified as shared hubs with docking scores of −10.800 kcal/mol and −6.445 kcal/mol, respectively. Curcumin-mediated inhibition of MEK/ERK signaling is well-documented [51], and Ediriweera et al. specifically demonstrated attenuation of leptin-induced ERK1/2 phosphorylation by Curcumin in BC cells, directly situating the MAP2K-ERK axis within the leptin-Curcumin interplay [35]. The predicted interaction with MAPK14 is consistent with Curcumin’s inhibition of p38 MAPK activation in inflammatory and BC contexts [29], though its specific role in leptin-stimulated cells remains to be defined. PTPN1 (PTP1B) was also identified as a shared hub, albeit with a docking score of −4.593 kcal/mol. Although classically considered a negative regulator of JAK2 in the hypothalamus, PTP1B acts as a positive regulator of oncogenic signaling in BC by activating Ras-MAPK signaling, specifically via p62 dephosphorylation [52].

Clinically, elevated PTPN1 expression is associated with poor prognosis, increased immune infiltration, heightened immune checkpoint activity, and reduced therapeutic sensitivity, whereas its knockdown enhances chemosensitivity in both MCF-7 and MDA-MB-231 cells [53]. It was previously reported that Curcumin inhibits PTP1B activity in these cells [54], implying that PTP1B suppression may contribute to Curcumin’s capacity to disrupt leptin-MAPK/ERK signaling. Curcumin also reduced STAT3 and STAT5 expression and phosphorylation, blocked STAT3 nuclear translocation, and inhibited its DNA-binding activity [34,51,55]. Although not identified as primary hubs, the JAK2/STAT3-STAT5 axis remains functionally significant, and RHOA-mediated attenuation of ROCK1/JAK2 may further dampen STAT3/STAT5 phosphorylation [7,9]. Because activation states, such as phospho-AKT, phospho-ERK, phospho-STAT3, and RHOA activity, were not directly measured, the proposed mechanistic model should be considered a hypothesis-generating model that requires further experimental validation.

Functional enrichment analyses revealed substantial overlap between MCF-7 and MDA-MB-231 cell lines in cytoskeleton and motility processes, driven by CDC42, RAC1, RHOA, AKT1, and PTPN1, highlighting actin remodeling, cell polarity, and migration as shared pathways co-regulated by leptin and Curcumin. Extracellular vesicle–associated components were also enriched, indicating vesicle-mediated intercellular communication. Cell line–specific patterns reflected subtype biology, with MCF-7 linked to epithelial organization and hormone-responsive pathways [56], whereas MDA-MB-231 showed enrichment in stress and invasion signaling. Shared enrichment in cytoskeletal regulation, vesicle biology, and AKT-centered signaling suggests a conserved vulnerability to Curcumin-mediated disruption of leptin signaling.

Integrating the experimental findings with network pharmacology and existing literature, we propose a hypothesis-generating model (Figure 9) in which the effects of Curcumin on leptin-associated oncogenic signaling may involve several candidate molecular nodes: attenuation of LepRb/JAK2 activation via RHOA interference; suppression of the PI3K/AKT/mTOR pathway through AKT1 and RHEB; inhibition of MAPK/ERK signaling via MAP2K1, MAPK14, and PTP1B; and blockade of the JAK2/STAT3-STAT5 axis. The predicted interactions identified by molecular docking provide computational support for the potential involvement of these proteins but do not establish direct binding or causal modulation of the corresponding signaling pathways. The proposed model therefore represents a testable framework for understanding the potential convergence of leptin-associated signaling and Curcumin responses rather than an experimentally established mechanism. Furthermore, several limitations should be considered. Leptin exposure models only one component of obesity-associated signaling and does not capture its endocrine, inflammatory, metabolic, and stromal complexity. In addition, this study relies on in vitro systems using a single cell line per subtype, which does not recapitulate the full complexity of the tumor microenvironment and the obese context. Also, network pharmacology and docking predictions require validation through functional (co-immunoprecipitation), proteomic (phosphoproteomic analyses) approaches, as well as any other valid experimental technique.

Curcumin represents a promising candidate for therapeutic applications; however, its poor aqueous solubility, limited intestinal absorption, instability under alkaline conditions, and rapid metabolism restrict its oral bioavailability and therapeutic potential. Several approaches have therefore been explored to overcome these limitations, including combination with piperine and delivery systems such as micelles, emulsions, nanoparticles, liposomes, and solid dispersions, with these strategies investigated in cell-based, animal, and human studies. Despite these challenges, Curcumin-based and nanoCurcumin formulations have shown promising clinical effects in cancer and ulcerative colitis, with several formulations demonstrating acceptable safety and tolerability. These findings support the potential clinical application of Curcumin, particularly through optimized formulations designed to enhance its bioavailability [57,58,59].

## 4. Materials and Methods

### 4.1. Cell Culture

MCF-7 and MDA-MB-231 cells were maintained in Dulbecco’s Modified Eagle’s Medium (DMEM high glucose W/O L-glutamine) (PAN-Biotech, Aidenbach, Germany) supplemented with 10% (*v*/*v*) fetal bovine serum, 1% L-glutamine, 100 units/mL of penicillin, and 100 µg/mL of streptomycin (PAN-Biotech), and incubated at 37 °C in a humidified atmosphere containing 5% CO_2_. The cells were enzymatically passaged using 0.05% trypsin-EDTA (PAN-Biotech) every 3 days until they reached approximately 80% confluency. For the assays conducted in this study, cells were cultured in high-glucose DMEM supplemented with 1% L-glutamine, 100 U/mL penicillin, and 100 µg/mL streptomycin, in the absence of FBS. Treatment conditions comprised exposing cells to non-cytotoxic Curcumin (Sigma-Aldrich C1386-10G, St. Louis, MO, USA) concentrations (5 and 10 µM), which were previously validated using an MTT assay. Cells were also treated with leptin (100 ng/mL) to mimic hyperleptinemic conditions, and this concentration was selected based on our previous findings [9].

### 4.2. Cell Viability Assay

MCF-7 and MDA-MB-231 cell lines were seeded into 96-well plates at a density of 7 × 10^3^ cells per well and incubated overnight for adherence. The cells were then treated with Curcumin (5, 10, 20, and 30 µM), 100 ng/mL of leptin, or the combination of Curcumin (5 and 10 µM) and leptin (100 ng/mL) for 48 h at 37 °C in a humidified atmosphere containing 5% CO_2_ saturation. Cell viability was assessed by applying MTT (3-(4,5-dimethylthiazol-2-yl)-2,5-diphenyl tetrazolium bromide) at a concentration of 0.5 mg/mL for 3 h at 37 °C. Formazan salts produced by viable cells were dissolved in dimethyl sulfoxide (DMSO), and the absorbance value was measured spectrophotometrically at 570 nm using a microplate reader.

The effect of leptin on cell proliferation in the presence or absence of Curcumin was assessed and expressed as the percentage of viable cells relative to leptin-treated control cells, which were considered 100% viable.

### 4.3. Wound-Healing Assay

Cell migration was analyzed using the wound-healing assay. Briefly, MCF-7 and MDA-MB-231 cells were seeded into 24-well plates at a density of 3.5 × 10^4^ cells per well in 500 μl of DMEM medium/10% FBS. After reaching confluence (90%), the medium was removed and replaced with DMEM without FBS to inhibit cell proliferation. After an overnight incubation, the cell monolayer was wounded by making a strip in the center of the well using a sterile 10 μl pipette tip. The medium was removed to eliminate the detached cells. Cells were then incubated with 500 µL of Curcumin (5 and 10 µM), leptin (100 ng/mL), or their combination, for 48 h at 37 °C in a humidified atmosphere containing 5% CO_2_. Cells incubated in DMEM medium were considered as control cultures. The rate of wound closure was assessed by taking one image of the wound’s center for every well at 0 and 48 h, using a light microscope (magnification, ×4 objective). Wound closure was quantified by measuring the area between the edges of the wound using the ImageJ software. Wound width was normalized to 100% at 0 h for each condition; data were expressed as percentages of wound closure.

### 4.4. 3D Tumor Spheroid Dissemination Assay

The ability of cells to disseminate from spheroids was evaluated using a modified protocol previously described. Spheroids were generated using the hanging-drop technique. Briefly, 2.0 × 10^3^ cells were resuspended in 17 µL of complete culture medium and were dispensed as individual drops at the bottom and center of each well in 24-well plates. The plate was then inverted and placed over a lid containing sterile water (¼ filled) to maintain humidity. Inverted plates were incubated at 37 °C with 5% CO_2_ for 4 days, until compact, spherical spheroids formed. After spheroid formation, the spheroids were incubated for 48 h in DMEM medium without FBS (vehicle), in vehicle supplemented with Curcumin alone (10 µM), recombinant human leptin alone (100 ng/mL), or in vehicle supplemented with leptin and Curcumin.

Images of the spheroids were acquired using an inverted microscope equipped with a digital camera at T0 and T48 h. Cell dissemination was quantified by measuring the total area occupied by cells surrounding the spheroid using ImageJ software. Measurements were obtained by manually outlining the boundaries of the dissemination zone at each time point. Data were expressed as the percentage increase relative to t0 and were calculated using the following formula:

Dissemination rate (%) = (STn/ST0) × 100

where STn is the area measured at T48 h, and ST0 is the initial area at T0.

### 4.5. Colony Formation Assay

MCF-7 and MDA-MB-231 cells were seeded into 6-well plates at a density of 2.0 × 10^3^ cells per well in 2 ml of DMEM medium/10% FBS. After an overnight incubation, cells were treated with Curcumin (5 and 10 µM), leptin (100 ng/mL), or their combination, and incubated for 48 h at 37 °C in a humidified atmosphere containing 5% CO_2_. Cells incubated in DMEM medium were considered as control cultures. The medium was removed and replaced with DMEM medium/10% FBS, and the cells were allowed to grow for up to 10 days and monitored until the formation of distinct visible colonies. The wells were then washed with phosphate-buffered saline (PBS), and cells were fixed with 4% paraformaldehyde and stained with 0.1% crystal violet diluted in water. Cell clones were counted using a light microscope, and only colonies containing a minimum of 50 cells were considered.

### 4.6. Statistical Analysis

All experiments were performed using three independent biological replicates (*n* = 3), and data are presented as mean ± standard deviation (SD). Statistical analyses were performed using GraphPad Prism version 8. Comparisons between two groups were performed using an unpaired Student’s *t*-test, whereas comparisons involving multiple groups were performed using one-way analysis of variance (ANOVA). A *p*-value < 0.05 was considered statistically significant.

### 4.7. Network Pharmacology Workflow for Curcumin–Leptin–BC Cell Lines

To investigate the molecular mechanisms through which curcumin may modulate leptin-driven BC, we employed a network pharmacology approach. Our analysis focused on two subtypes represented by MCF-7 (Luminal A) and MDA-MB-231 (TNBC) cell lines. In silico platforms were used to predict Curcumin-related molecular targets, while leptin and BC-associated gene sets were obtained from experimentally characterized cell line datasets. These data were then combined for network construction, hub gene identification, functional enrichment analysis, and molecular docking.

#### 4.7.1. Retrieval of Curcumin, Leptin Targets and Cancer Cell Line–Associated Genes

Curcumin’s structure was retrieved from the PubChem database in SDF format and submitted to the PharmMapper server (http://www.lilab-ecust.cn/pharmmapper/, accessed on 23 November 2025) for target prediction. All predicted targets were standardized using the UniProt ID mapping tool (https://www.uniprot.org/id-mapping, accessed on 23 November 2025). Genes associated with leptin signaling were retrieved by querying “Leptin” in the GeneCards database (https://www.genecards.org/, accessed on 23 November 2025) [60]. Only protein-coding genes with a relevance score ≥ 1 were retained [61]. Specific gene expression data of breast cancer cells were obtained from the DepMap 25Q2 release (https://depmap.org/portal/, accessed on 23 November 2025) using the “DepMap 25Q2 TPMLogp1” datasets, which provide normalized transcript abundance values for protein-coding genes. For each model, in MCF-7 (DepMap ID: ACH-000019) and MDA-MB-231 (DepMap ID: ACH-000768), protein-coding genes were ranked according to their normalized expression values in the dataset. The top 2000 expressed genes were retained for subsequent target intersection. This cutoff was selected based on comparison of alternative expression-rank thresholds and provided a balance between retaining highly expressed genes and maintaining adequate target coverage.

#### 4.7.2. Protein–Protein Interaction (PPI) Network Construction and Hub Gene Identification

The overlapping targets between Curcumin and the disease-associated genes were identified using Venn Diagram Online Tool v2.1.0 (https://bioinfogp.cnb.csic.es/tools/venny/index.html, accessed on 23 November 2025). These common targets were used to construct a protein–protein interaction (PPI) network using the STRING database (https://string-db.org/, accessed on 23 November 2025) [62], with *Homo sapiens* selected as the organism and a minimum required interaction score of 0.4 (medium confidence) [61,62,63].

This threshold was selected to preserve biologically meaningful network connectivity essential for systems-level network pharmacology analysis, particularly following prior TPM-based transcriptomic filtering, which minimizes spurious interactions while enabling robust identification of key targets and pathways. The topological parameters of the PPI network were analyzed using Cytoscape v3.10.4. The degree of interaction for each node was calculated, enabling the identification of the hub genes with the highest connectivity, highlighting their potential central role in disease modulation. Following this, the PPI network was visualized in Cytoscape to provide an intuitive representation of the interactions among Curcumin targets and disease-related genes.

#### 4.7.3. Gene Ontology (GO) and Pathway Enrichment Analysis

To conduct the functional enrichment analysis for Biological Process (BP), the latest version of ShinyGO (v0.85.1) (https://bioinformatics.sdstate.edu/go/, accessed on 23 November 2025) by GO was used. Enrichment tables and plots were generated to provide a clear overview of the main biological processes associated with the shared targets.

#### 4.7.4. Molecular Docking Simulation, Validation and Estimation of Binding Free Energy (MM-GBSA)

Molecular docking simulations were performed using Schrödinger Maestro 12.8 (Release 2021-2). Three-dimensional crystal structures of the druggable proteins encoded by the ten identified hub genes were retrieved from the Protein Data Bank (PDB). Only structures with a resolution better than 2.5 Å were selected for subsequent analyses. Protein structures were prepared using the Protein Preparation Wizard by adding hydrogen atoms, correcting missing residues where necessary, assigning appropriate protonation states under neutral pH conditions, and subsequently performing energy minimization. Receptor grids were generated in Maestro, with the binding sites defined according to the positions of the corresponding co-crystallized ligands. Curcumin was used as the sole ligand and was retrieved from PubChem. Ligand preparation was performed using the LigPrep module. The ligand was processed with ionization set to neutral, while the default tautomeric and desalt states were used during ligand preparation, followed by energy minimization using the OPLS3e force field. The prepared ligand states were subsequently used for molecular docking. Molecular docking was performed using Glide in Extra Precision (XP) mode [64]. The resulting docking scores and corresponding binding poses were recorded for the Curcumin–protein complexes. The docking protocol was evaluated by redocking the corresponding co-crystallized ligand into its binding site, and the root mean square deviation (RMSD) between the experimentally observed and redocked ligand poses was calculated as a measure of the ability of the docking protocol to reproduce the known binding orientation. The binding free energies of the ligand–protein complexes were estimated using the Molecular Mechanics/Generalized Born Surface Area (MM-GBSA) method. Based on the energetic contributions of the free ligand, free receptor, and receptor–ligand complex, the MM-GBSA method was used to estimate the binding free energies of the selected protein–ligand complexes [65]. Following molecular docking, selected top-ranked complexes were subjected to MM-GBSA analysis, and the binding free energy (ΔG_bind_) was calculated using the equation:

ΔG_bind_ = G_complex_ − (G_protein_ + G_ligand_)

where G_complex_ is the free energy of the complex, ΔG_bind_ is the binding free energy of the protein–ligand complex, G_protein_ is the free energy of the protein in the absence of the ligand, and G_ligand_ is the free energy of the ligand in the absence of the protein. The ligand–protein complexes were visualized using Discovery Studio 25 Client.

## 5. Conclusions

In conclusion, the study suggests that Curcumin attenuates the pro-tumorigenic effects of leptin, reducing cell proliferation, clonogenicity, and migratory capacity, in both luminal A and triple-negative BC cell lines. By integrating network pharmacology with existing bibliographic data, we identified RHOA, AKT1, RHEB, MAP2K1, MAPK14, and PTPN1 as candidate convergence nodes within the leptin-Curcumin signaling interplay and propose a mechanistic model linking these hub genes to the observed phenotypic outcomes. These findings address a previously underexplored dimension of the leptin-Curcumin axis in BC, especially in the obesity context. Future work should prioritize the functional validation of the identified hub proteins and strengthen the proposed signaling model linking leptin and Curcumin responses. Such studies will be essential to confirming causality, delineating pathway-specific contributions, and ultimately translating this multitarget framework into adjuvant nutraceutical therapeutic strategies for obesity-associated BC.

## Figures and Tables

**Figure 1:**
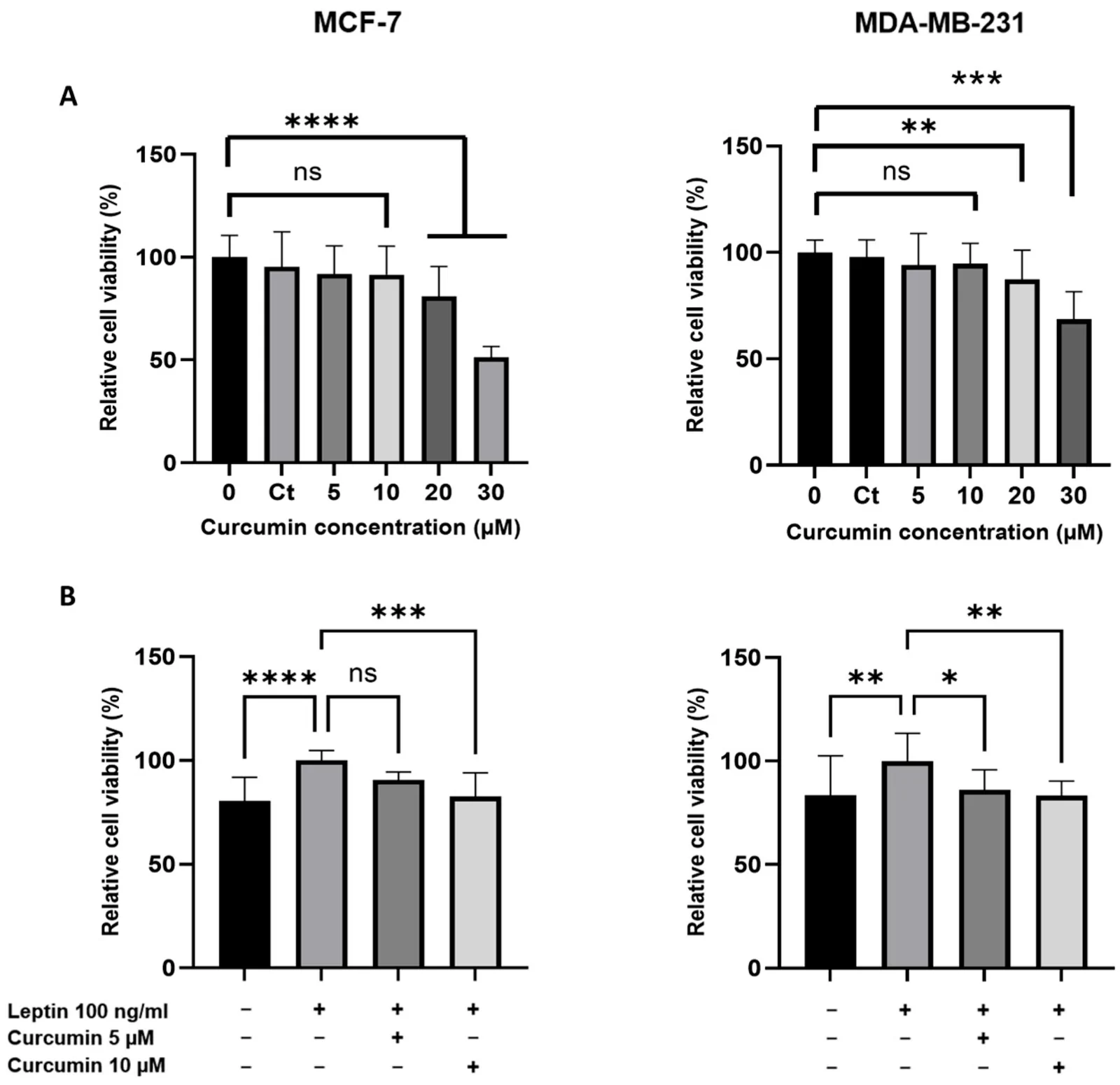
Curcumin suppresses leptin-mediated viability in BC cells. (**A**) MCF-7 and MDA-MB-231 cells were treated with increasing concentrations of Curcumin (5, 10, 20, and 30 µM) for 48 h. Untreated cells (0 µM) and cells exposed to 0.1% DMSO (Ct) served as controls. (**B**) Cells were treated with leptin (100 ng/mL), alone or in combination with Curcumin (5 or 10 µM), for 48 h. Cell viability was assessed by MTT assay and expressed as relative viability (%). Data are presented as mean ± SD from at least three independent biological replicates. Statistical significance was assessed by one-way ANOVA: * *p* < 0.05, ** *p* < 0.01, *** *p* < 0.001, **** *p* < 0.0001; ns, not significant.

**Figure 2:**
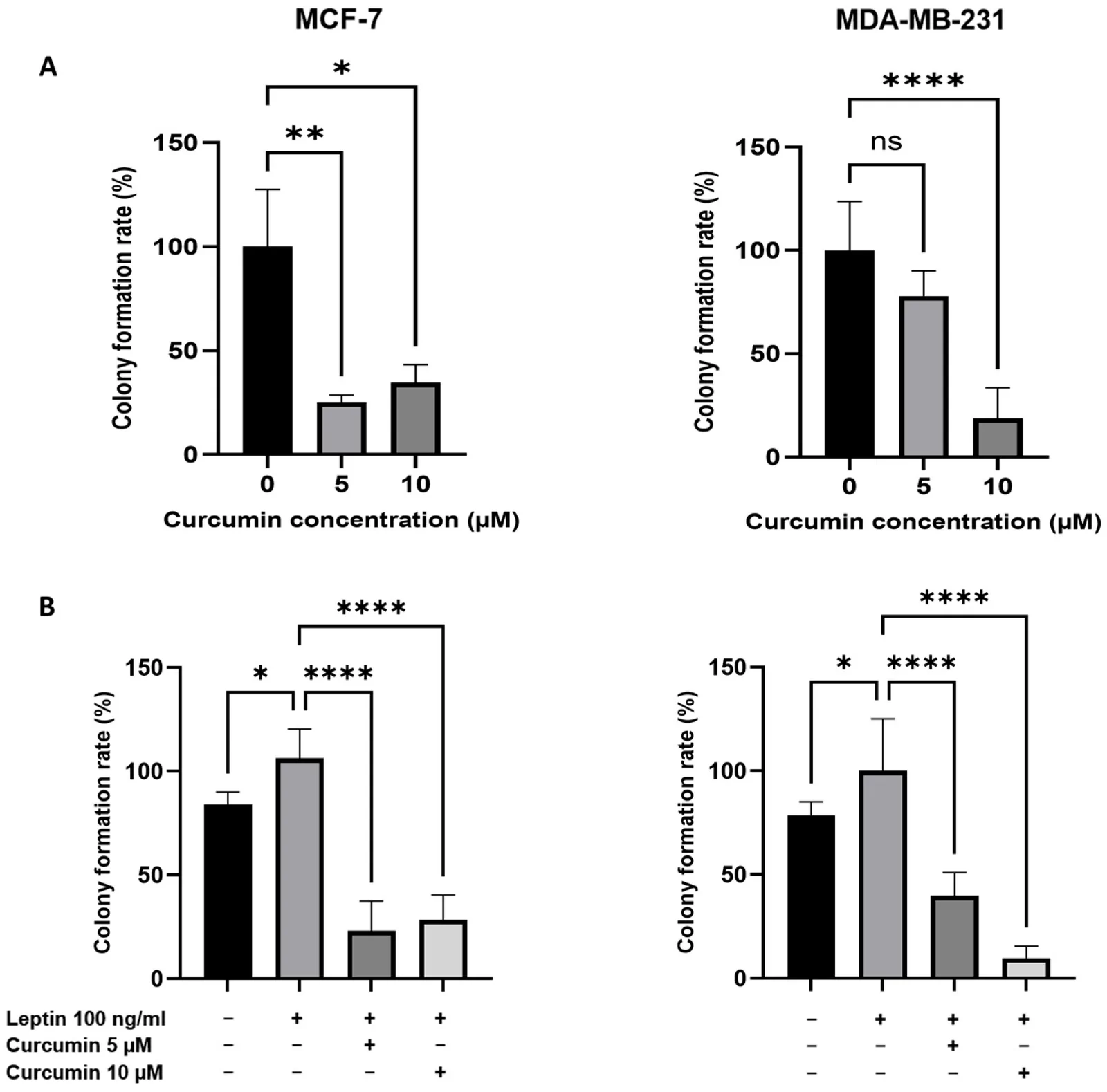
Curcumin inhibits leptin-induced clonogenic potential in BC cells. (**A**) MCF-7 and MDA-MB-231 cells were treated with Curcumin (5 or 10 µM) for 48 h to assess its effect on colony formation. (**B**) Cells were treated with leptin (100 ng/mL), alone or combined with Curcumin (5 or 10 µM), for 48 h. Following treatment, cells were cultured for 10 days, and colonies containing more than 50 cells were stained with crystal violet and counted. Colony formation rates are expressed relative to the untreated control. Data are presented as mean ± SD from at least three independent biological replicates. Statistical significance was assessed by one-way ANOVA: * *p* < 0.05, ** *p* < 0.01, **** *p* < 0.0001; ns, not significant.

**Figure 3:**
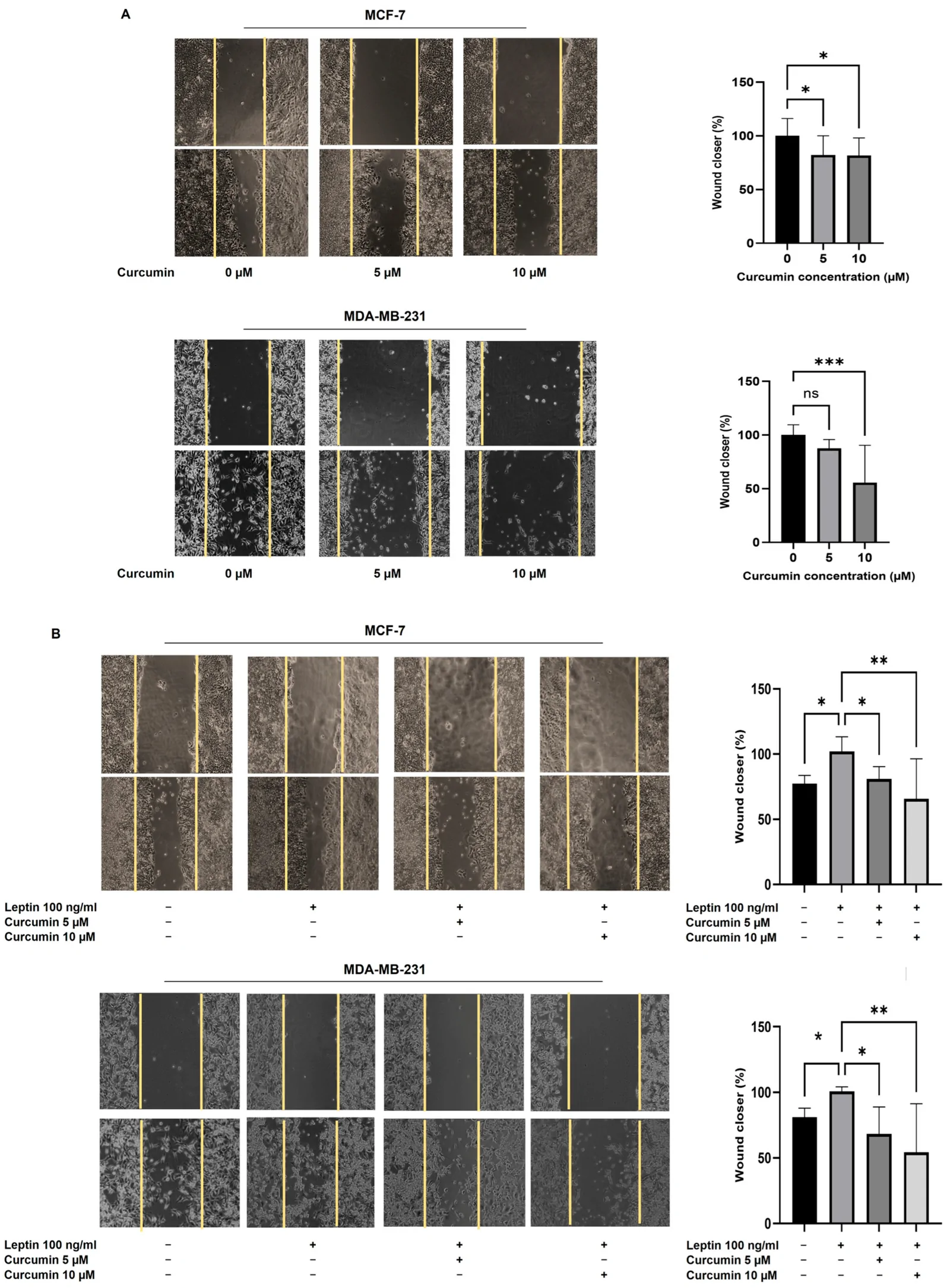
Curcumin inhibits leptin-induced migration of BC cells. (**A**) MCF-7 and MDA-MB-231 cells were treated with Curcumin (5 or 10 µM) for 48 h. (**B**) Cells were treated with leptin (100 ng/mL) alone or in combination with Curcumin (5 or 10 µM) for 48 h. Cell migration was assessed using a wound-healing assay, and wound closure was quantified using ImageJ (version 1.54). Representative images are shown alongside the corresponding quantification. Data are expressed as relative wound closure (%) and presented as mean ± SD from at least three independent biological replicates. Statistical significance was determined by one-way ANOVA: * *p* < 0.05, ** *p* < 0.01, *** *p* < 0.001; ns, not significant.

**Figure 4:**
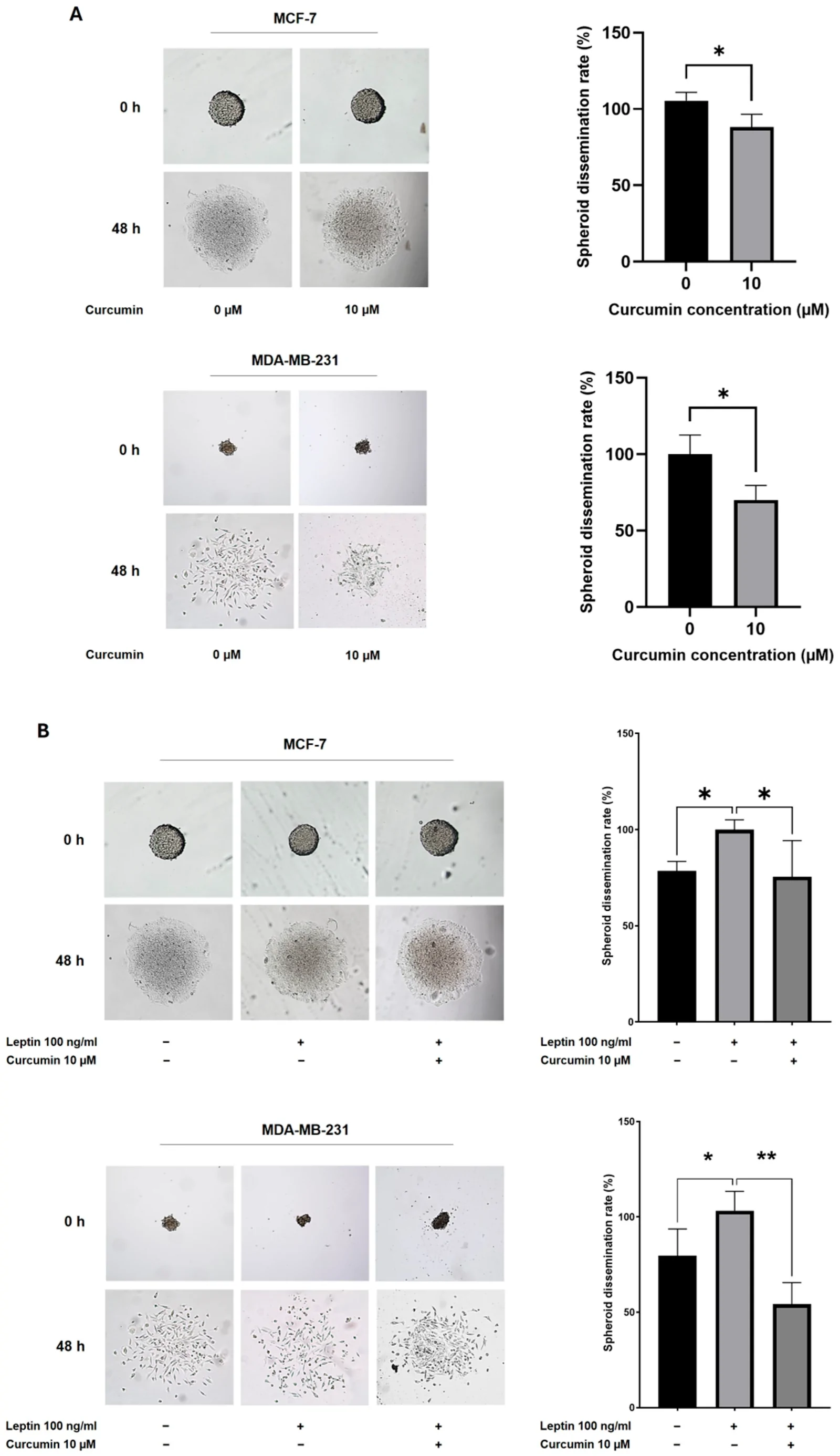
Curcumin attenuates leptin-induced 3D spheroid dissemination in BC cells. (**A**) MCF-7 and MDA-MB-231 spheroids were treated with Curcumin (10 µM) for 48 h. (**B**) Spheroids were treated with leptin (100 ng/mL), alone or in combination with Curcumin (10 µM), for 48 h. Spheroid dissemination was assessed from representative images acquired at T0 and T48 h, and the increase in the area surrounding each spheroid was quantified using ImageJ. Data are expressed as relative dissemination rate compared with the leptin-treated control and presented as mean ± SD from at least three independent biological replicates. Statistical analysis was performed using unpaired *t*-tests for (**A**) and one-way ANOVA for (**B**): * *p* < 0.05, ** *p* < 0.01.

**Figure 5:**
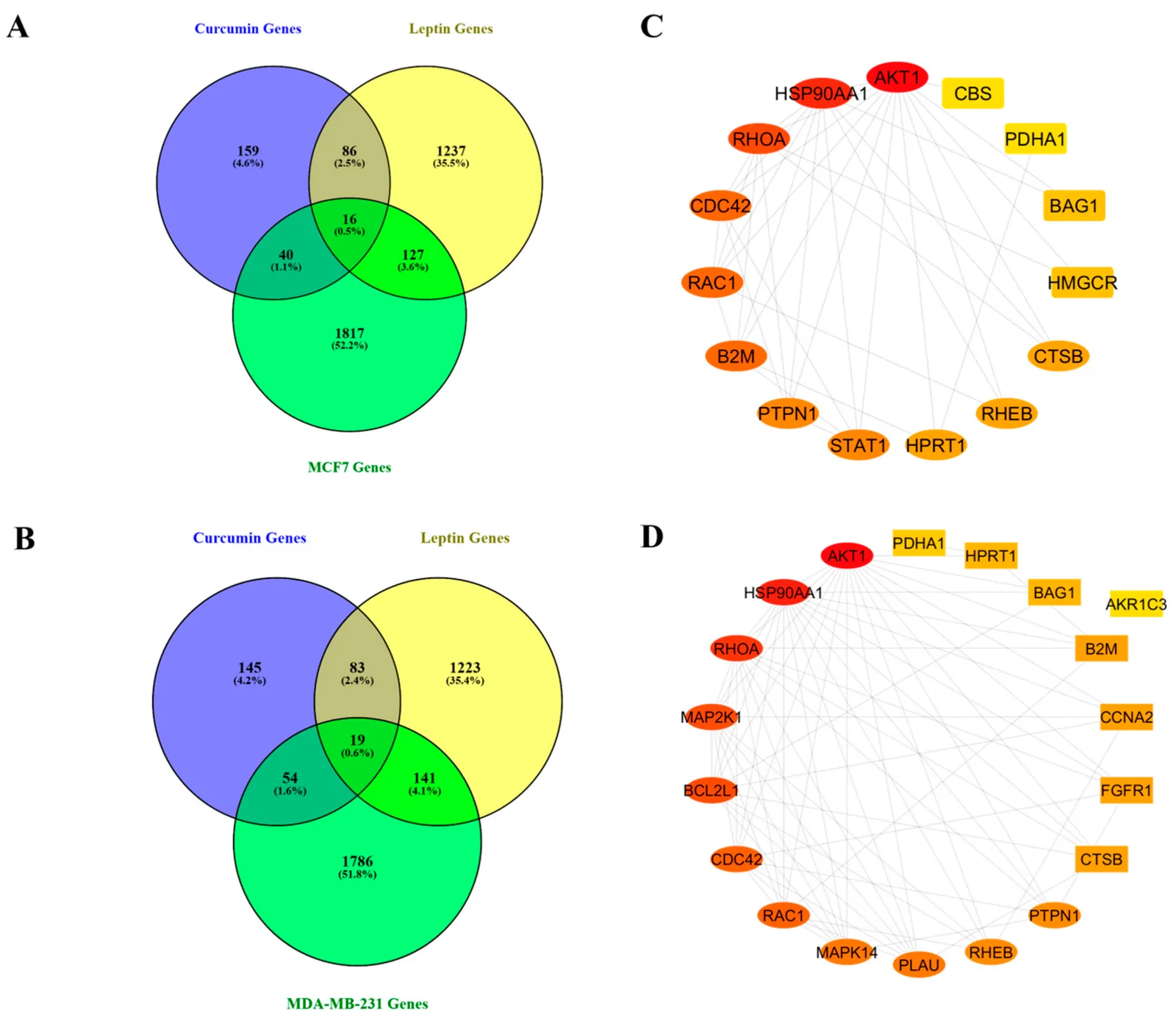
Network-based analysis identifies conserved and subtype-specific hub targets shared by Curcumin and leptin in BC cells. Putative protein-coding targets were retrieved and overlaps visualized by Venn diagrams revealing 16 shared targets in MCF-7 (**A**) and 19 in MDA-MB-231 (**B**) cells. Common targets were used to construct STRING-derived PPI networks, followed by topological analysis in Cytoscape (**C**,**D**, corresponding to MCF-7 and MDA-MB-231, respectively). Hub genes, defined by high interaction degree, are shown as ellipses, whereas non-hub nodes are rectangles. Node color intensity indicates connectivity, with red representing the highest degree.

**Figure 6:**
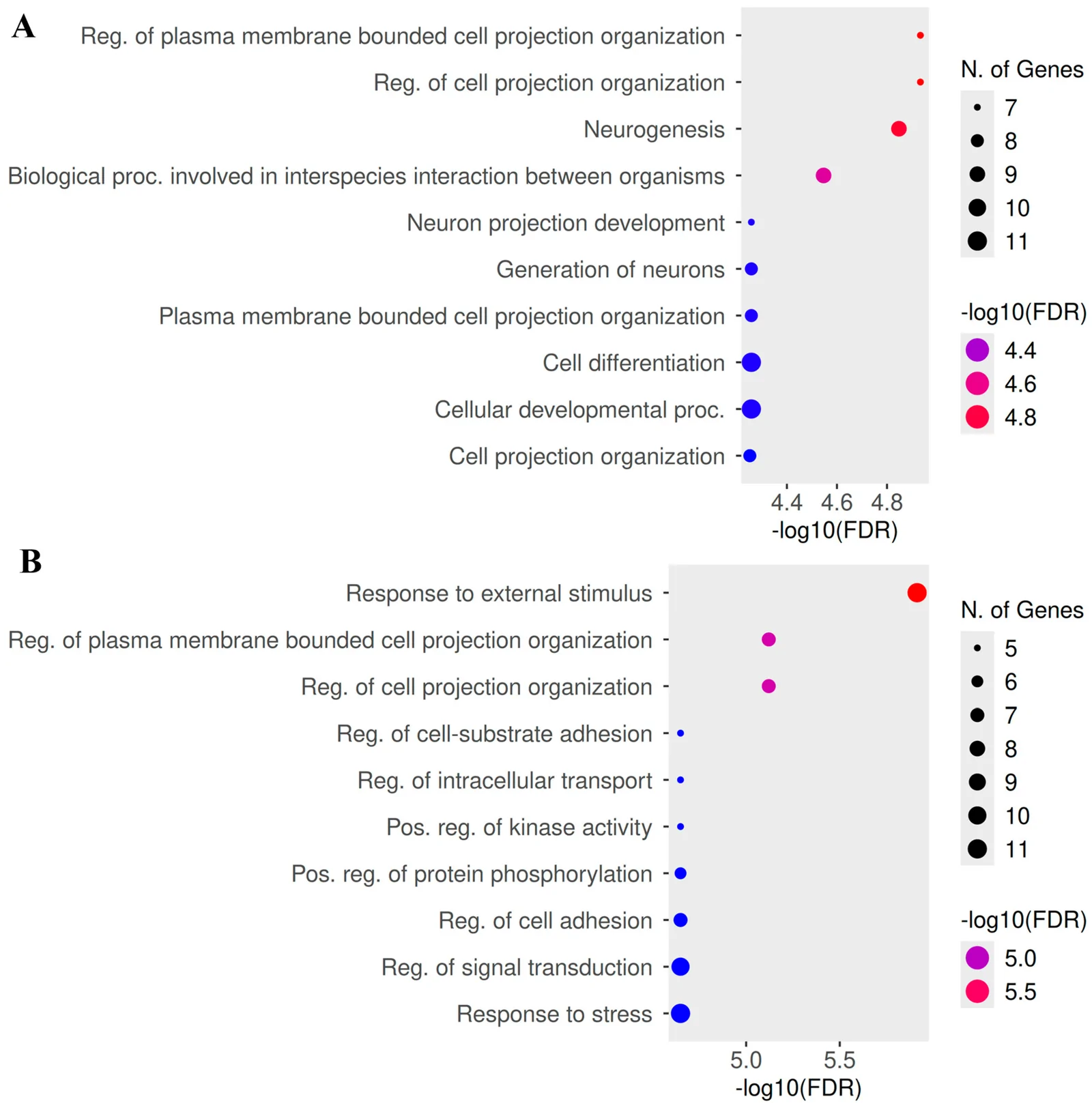
Functional enrichment analysis of shared Curcumin–leptin targets highlights key biological processes in BC cells. Gene Ontology enrichment was performed using ShinyGO for common targets in MCF-7 (**A**) and MDA-MB-231 (**B**) models. Enrichment plots display significantly overrepresented biological processes, ranked by statistical significance. Dot size indicates gene count per term, while the color gradient reflects enrichment significance level.

**Figure 7:**
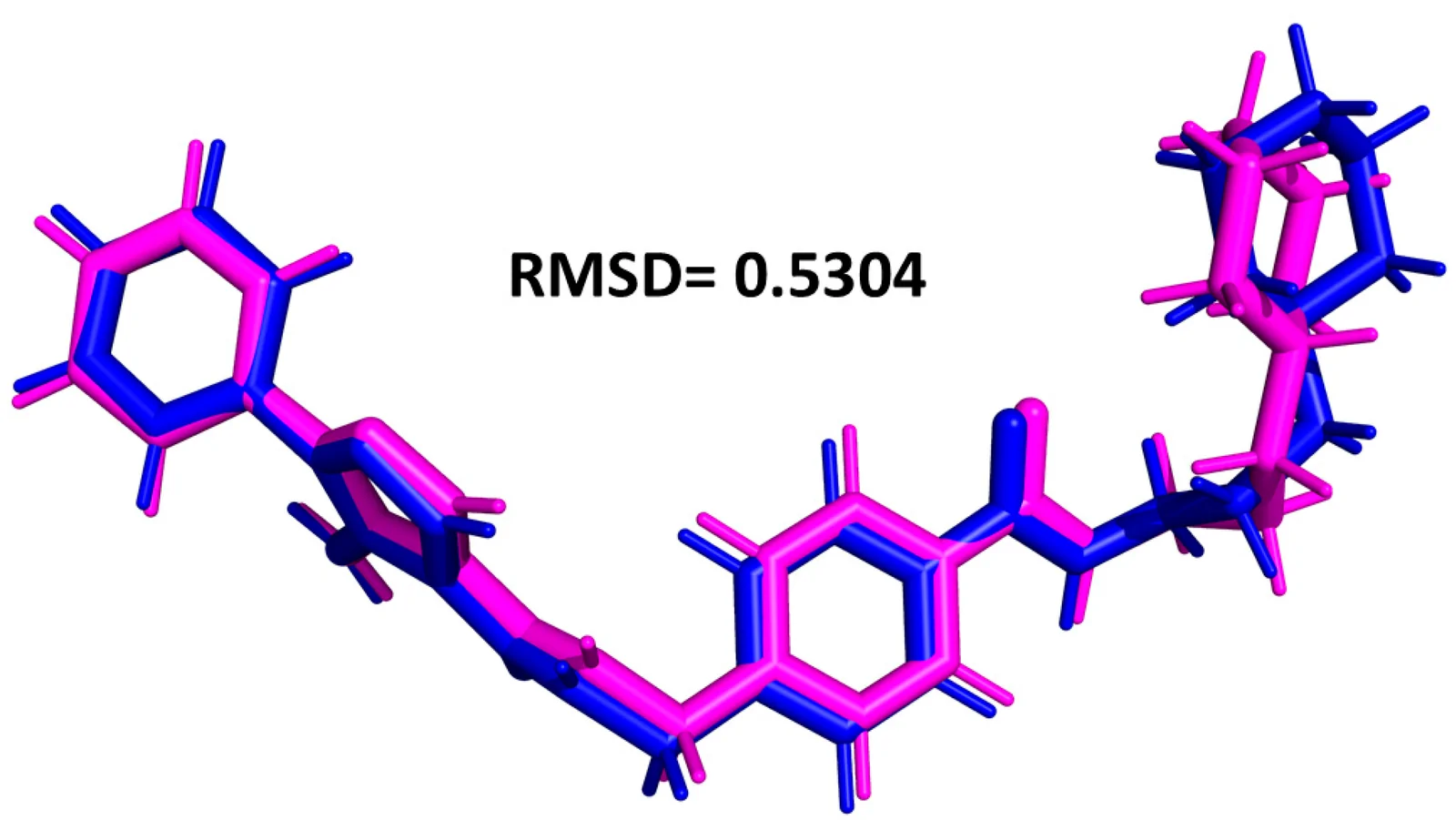
Superposition of the co-crystallized ligand (blue) and the redocked pose (magenta), yielding an RMSD of 0.5304 Å. The low RMSD value indicates a close agreement between the experimental and redocked ligand conformations, supporting the reliability of the docking protocol.

**Figure 8:**
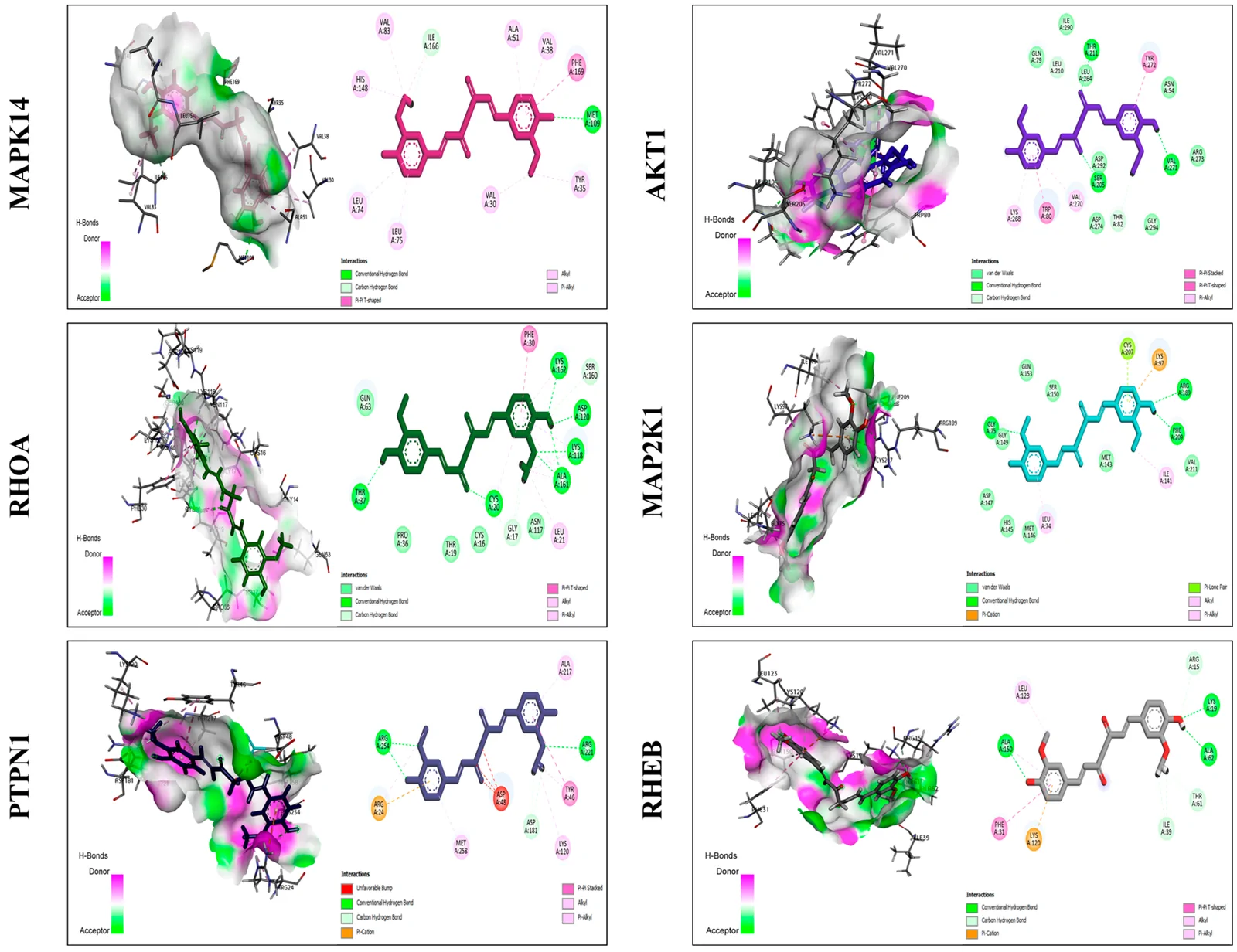
Three-dimensional (3D) and two-dimensional (2D) visualization of Curcumin–protein docking complexes reveals stable, high-affinity interactions with leptin-associated oncogenic hub proteins in BC cells. Representative complexes with MAPK14, AKT1, RHOA, MAP2K1, PTPN1, and RHEB are shown. The 3D views (**left**) depict Curcumin positioning within binding pockets, highlighting hydrogen-bond donor (magenta) and acceptor (green) regions, as well as surface complementarity. Corresponding 2D diagrams (**right**) detail residue-level interactions, supporting strong binding stability. Visualizations were generated using Discovery Studio 25 Client.

**Figure 9:**
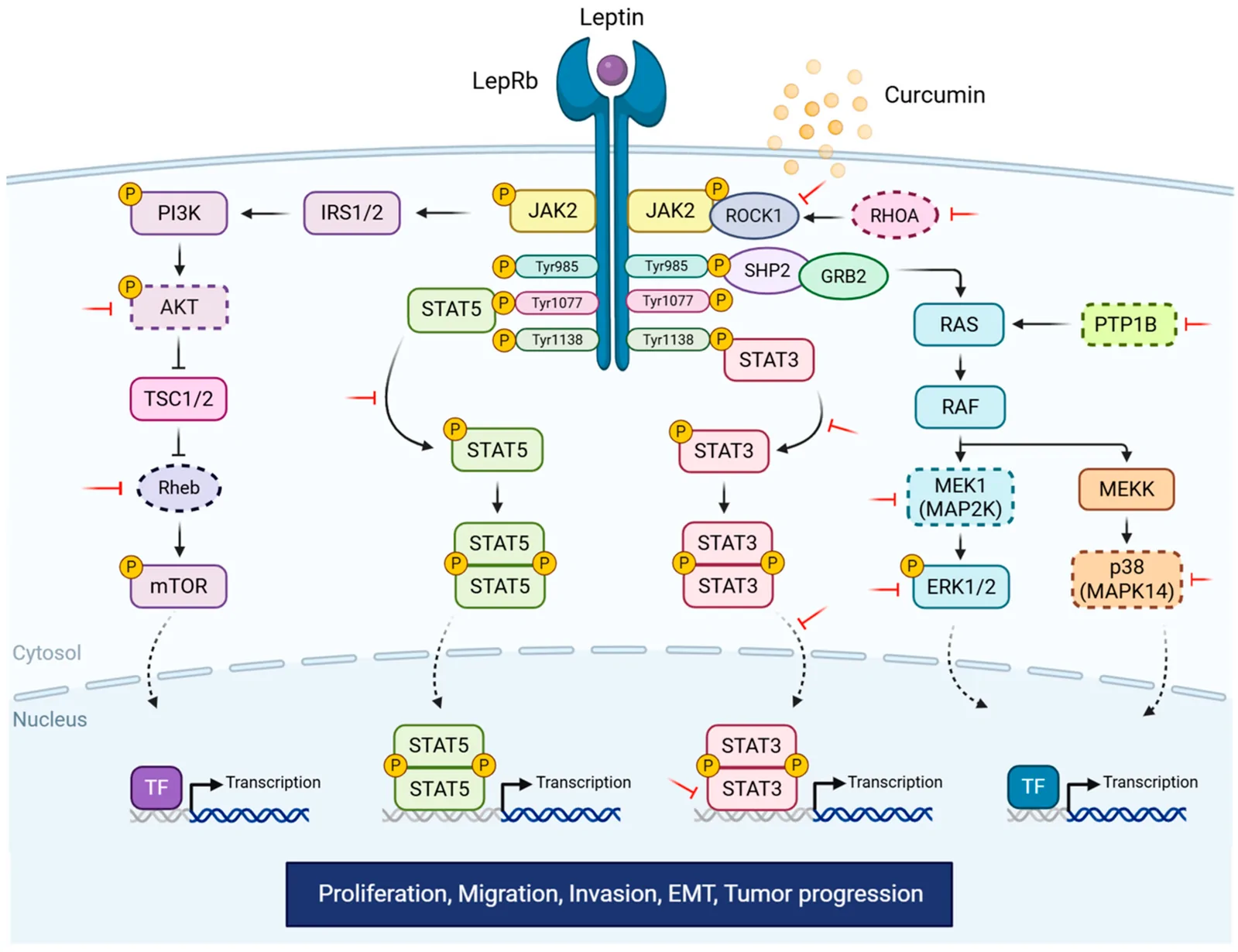
Proposed hypothetical crosstalk between leptin and Curcumin signaling in BC cells based on literature evidence and pharmacology network analysis. Leptin binding to the LepRb promotes recruitment of ROCK1 and interaction with GTP-bound RhoA, activating RhoA/ROCK and JAK2 signaling. JAK2 phosphorylates Tyr985, Tyr1077, and Tyr1138 within the intracellular domain of LepRb, generating docking sites that coordinate activation of JAK2/STAT3–STAT5, MAPK/ERK–JNK, and IRS–PI3K–AKT–mTORC1 pathways. IRS–PI3K–AKT signaling inhibits TSC1/2, enabling RHEB-mediated activation of mTORC1 and protein synthesis. Phosphorylation of Tyr985 leads to activation of SHP2 and downstream MAPK and ERK1/2 cascades, while phosphorylation at Tyr1138 promotes STAT3/STAT5 phosphorylation and nuclear translocation and signaling. PTPN1 (PTP1B) links leptin signaling to Ras–MAPK pathways, enhancing tumorigenesis by activating Ras GTPases. Dashed arrows indicate nuclear translocation. Hub proteins identified by pharmacology network analysis are indicated by dashed borders. Red bars indicate inhibition by Curcumin.

**Table 1:** Docking scores and MM-GBSA binding free energies of Curcumin with selected hub proteins identified in BC cells. Docking was performed using Glide in Extra Precision (XP) mode, and the protocol was validated by redocking the native co-crystallized ligand, with root mean square deviation (RMSD) values calculated to confirm docking reliability. Binding free energies were estimated using the MM-GBSA approach, which integrates the energetic contributions of the free ligand, free receptor, and receptor–ligand complex to assess interaction stability. The table summarizes docking scores and MM-GBSA ΔG values.

Gene	PDB ID	Glide Docking Score (kcal/mol)	MM-GBSA ΔG_bind (kcal/mol)
*MAPK14*	6SFO	−10.80	−63.10
*AKT1*	8UW7	−8.285	−66.95
*RHOA*	1FTN	−7.107	−55.41
*MAP2K1*	8YP4	−6.445	−58.10
*PTPN1*	1PTY	−4.593	−48.37
*RHEB*	1XTQ	−4.475	−48.43

## Data Availability

The original contributions presented in this study are included in the article. Further inquiries can be directed to the corresponding authors.
